# Socioeconomic and demographic determinants of familial social capital inequalities: a cross-sectional study of young people in sub-Saharan African context

**DOI:** 10.1186/s12889-020-09135-0

**Published:** 2020-06-22

**Authors:** Evelyn Aboagye Addae

**Affiliations:** Department of Sociology and Social Policy, Lingnan University, 8 Castle Peak Road, Tuen Mun, Hong Kong

**Keywords:** Familial social capital, Socioeconomic and demographic determinants, Social capital inequalities, Family sense of belonging, Family autonomy support and control, sub-Saharan African context, Young people

## Abstract

**Background:**

Social capital is broadly acknowledged as a vital ‘health asset’ that promotes young people’s health and wellbeing and has the potential to prevent social- and health-related risk behaviours in the life-course. However, limited research has investigated the determinants of social capital for young people in sub-Saharan Africa. This study examines the role of socioeconomic and demographic factors in establishing inequalities in familial social capital among young people in Ghana.

**Methods:**

The study utilised a cross-sectional survey data involving 2068 in-school adolescents (13-18 years) in the Upper West Region, Ghana. Familial social capital was assessed by ‘family sense of belonging’, ‘family autonomy support’ and ‘family control’. Multinomial logistic regressions established the relationships between socioeconomic and demographic factors and the measures of familial social capital.

**Results:**

Adolescents from low affluence households had about 63 and 61% lower odds of attaining a high family sense of belonging (FSB) (OR = 0.373; 95%CI: 0.27–0.513) and high family autonomy support (FAS) (OR = 0.387; 95%CI: 0.270–0.556) respectively but had 67% higher odds of reporting high family control (FC) (OR = 1.673; 95%CI: 1.187–2.359) than their counterparts. Males had about 55 and 71% higher odds to possess high FSB (OR = 1.549; 95%CI: 1.210–1.983) and high FAS (OR = 1.705; 95%CI: 1.272–2.284) respectively but had 38% lower odds to report high family control (OR = 0.624; 95%CI: 0.474–0.822) than females. The odd of young adolescents to attain high FSB than older adolescents were about 66% higher (OR = 1.662; 95%CI: 1.168–2.367). Religion, educational level, ethnicity, family structure, and marital status were also significant predictors of adolescents’ family sense of belonging, family autonomy support and, family control.

**Conclusions:**

Socioeconomic and demographic factors influence inequalities in the amount of familial social capital possessed by young people which suggests possible risks of social inequality. The family context is possibly failing some cohorts of young people with particular reference to female and poor adolescents regarding familial cognitive social capital. Public health strategies should include families in addressing socioeconomic and demographic differences in social capital with a key focus on the cohorts of young people at risk of social capital inequality.

## Background

Translating social capital to the health and wellbeing promotion of young people has in recent years gained great consideration among some scholars and policy practitioners within the public health arena, particularly from some developed countries such as the United Kingdom [1–5]. Studies show that social capital is potentially considered as a ‘health asset’ for young people [2]. Thus, social capital can function as a resource that boosts the capability of young people to maintain and preserve health and wellbeing [2, 6]. For instance, evidence from WHO-Health Behaviour in School-aged Children studies revealed that social capital matters for young people’s health and wellbeing [1, 3]. In these studies, several social capital indicators including family, school, and neighbourhood sense of belonging were found to be related to the wellbeing, health, and health-promoting behaviours of young people in England and Spain [1, 3]. There are also indications that social approaches to the management and delivery of public health have substantial capability to enhance health, especially, for socially deprived individuals [5, 7]. Subsequently, in recent years, strategies aiming to tackle the social determinants of health, comprising social connectedness, have risen significantly [5]. Examples of such strategies include the establishment of community engagement/health initiatives and social prescribing [8, 9]. Examples of interventions employed in Scotland in the early years include the universal health visiting service, the Family Nurse Partnership, and the Triple P Positive Parenting Programme [5, 10]. Also, in England where knife violence is recently recognised as a public health concern, social approaches/interventions to combating knife violence include promoting positive parent-child relationships, promoting school and community social support for school children, and promoting future role models for school children [11]. Moreover, many social-related adult health behaviours that underpin health and wellbeing inequalities such as depression and anxiety have been found to begin at the early stages of life especially during adolescence [12, 13]. Employing social approaches to promoting health and wellbeing for young people could hence conceivably help to counteract several health-related problems in adulthood. Fostering social capital for the health and wellbeing promotion of young people in sub-Saharan Africa is, therefore, critical. While social capital has been defined from different perspectives, the common idea suggests the *interactions between members which enable the creation and preservation of valuable social resource, and the significance of positive social networks of diverse types and amounts that promote social development and health between diverse groups and societies* [14–16]. Social capital can either be employed at the individual or collective level. At the individual level, social capital is perceived to consist of people’s social relationships within their social environment such as the family from which they can accumulate resources for their benefits [17]. Social capital consists of structural dimension (the extent of social relationships such as social participation) and cognitive dimension (understanding the features of social relationships such as trust, sense of belonging, autonomy, and control) [18]. There has been an emergent approach of translating social capital into public health from the perspective of ‘health asset’ for young people [3]. Health assets are defined to augments the capability of individuals, societies, and populaces to preserve and uphold health and wellbeing [6]. The *asset approach* suggests that young people should be provided with social capital earlier in their lives [2]. Thus, aside from the family context, health assets can be attained by young people from members within their social environments to promote their health and wellbeing. The *asset approach* proposes that as much as social capital and its associated constructs are supported at the early stage of life, young people can experience more positive impacts from members within their social environment (families, school, local neighbourhoods, and communities) [2]. This notion also portrays young people as *social agents* that can contribute to shaping their own social lives and that of others in their societies by maximising health assets and minimising risks to promote wellbeing outcomes [3]. Young people can hence accumulate numerous social resources from their networks to promote prospects for high health and wellbeing. Moreover, the *asset approach* seeks active youth involvement, promoting a sense of belonging and feelings of autonomy and control to empower young people with credence and disposition to engage in various kinds of networks [2, 3]. Identifying and addressing the determinants of young people’s ‘health assets’ is, therefore, crucial for the current and future health promotions of any populace. Generally, social capital has been related to the health and wellbeing outcomes of young people [1–5, 19–21]. Evidence suggests that young people with a high amount of social capital (parent-child relationship, family sense of belonging, peer relationship, neighbourhood social capital, community social capital, school sense of belonging, family and school social and autonomy support) report higher health and wellbeing benefits (life satisfaction, happiness, psychological wellbeing, and self-reported health) than those with low amount of social capital [3, 4, 19–21]. For example, young people with low sense of family, school and neighbourhood sense of belonging are more likely to report poor health, low happiness and low life satisfaction [1, 3, 19–21]. On the other hand, socioeconomic [21–24] and demographic factors such as age, gender, race, religion, family structure, educational level, type of region, etc. [25–27] have been found to affect individual’s amount of social capital. According to studies from Malaysia [22] and from China [20], educational level and employment status affect children’s familial social capital (parental involvement and parent-child relationship). Hagan [23] and Huang et al. [24] also found that socioeconomic status is associated with familial social capital (parent-child relationship, parental expectations, and abundant family resources). King et al. [25] and King & Boyd [26] in the United States independently revealed that age, gender, race, religion, and family structure affect adolescent’s level of family connectedness which is an indicator of familial social capital. Additionally, using an adult population, a study done in Australia revealed a direct relationship between age, sex, household income, educational level, health status, and social capital [27]. Moreover, Nieminen et al. [28] found in Finland that the amount of one’s social capital is affected by age, income, marital status, type of region (urban/rural), and educational level. Other studies show variations in trust by gender, age, partnership, and education [29, 30]. Social capital has again been found to associate positively with age, income, and children, and negatively with town size and individualism in a European study [31]. These pieces of evidence clearly show that not all segments of a population possess equal amounts of social capital. However, efforts and early interventions to identify socioeconomic and demographic determinants of social capital can provide insight into how social inequality is established within societies particularly among young people. If social capital is vital for promoting health, then the determinants of social capital, especially at the early stages of life, are equally vital for indirectly promoting health through social capital. Nevertheless, few studies exist on the socioeconomic and demographic inequalities in social capital for young people in Ghana and sub-Saharan Africa largely. Most existing studies also focus on trust and structural social capital such as the number of social networks, civic engagement, and club participation. There is, therefore, scarce literature on socioeconomic and demographic related inequalities in familial social capital more evidently in the sub-Saharan African context.

A study by Adaae [21] provided evidence on the role of familial social capital as a potential health asset for the life satisfaction and happiness of adolescents in the sub-Saharan African context. This study revealed social capital within the home context to be a more significant protective health asset for the life satisfaction and happiness of Ghanaian adolescents against the effects of socioeconomic status than community (school) social capital. This finding provides the groundwork for why this presence study proposes that familial social capital of young people in sub-Saharan Africa particularly, Ghana should be considered and promoted in public health strategies and research. To effectively protect and promote young people’s accumulation of familial social capital in Ghana and promote the role of the family in constructing social capital, the determinants of familial social capital need to be recognised to provide targeted public health strategies for young people who are being left behind. This present study is hence related to and a contribution to the study by Addae [21] to fill the gap in the study of which the independent contributions of socioeconomic and demographic factors to Ghanaian adolescents’ social capital could not be examined for targeted policy recommendations. The examination of the determinants of social capital would provide potential insight into how social capital is distributed among various segments of Ghanaian adolescents in relation to the home context and consequently, safeguard the building of familial social capital for the wellbeing of Ghanaian young people. The present study, therefore, examined the roles of socioeconomic and demographic factors in establishing inequalities in young people’s familial social capital in a sub-Saharan African context using the same Ghanaian adolescent sample and data employed by Addae [21]. This was achieved by examining the interplay among several indicators of adolescents’ socioeconomic and demographic factors and their familial social capital simultaneously.

## Methods

### Sample and design

This study employed data obtained from a school-based cross-sectional survey carried out in 2018 on the health and wellbeing of young people in Ghana. The aim of the study was to examine the interplay among socioeconomic status, social capital, and the health and wellbeing of Ghanaian young people. The study involved 2068 adolescents aged 13–18 years randomly selected using a multi-stage stratified sampling approach from 7 districts in Ghana’s Upper West Region. First, primary sampling units consisting of three sub-regional zones (low, medium, and high poverty zone) were created on the bases of their unique sociodemographic, sociocultural factors, and poverty index. Based on the Ghana poverty mapping, from each of the low and high poverty incidence zones, two districts were randomly selected. From the medium poverty zone, three districts were selected because it had the largest number of districts. This approach was to provide equal opportunity for any of the districts to be chosen. This study employed only male-and female-mixed public schools to ensure sample homogeneity. This is because almost all the secondary schools available at the time of the study were mixed public schools. For the basic school level, Junior High Schools were included. Using simple random sampling, one Junior High School and one Senior High School were, therefore, selected from each district to represent the sample units. However, for one district, two Senior High Schools were selected due to the limited sample size. This was to ensure a representative sample size for all the zones created. A total of fifteen schools comprising eight Senior High Schools and seven Junior High Schools were, therefore, employed. Students below 13 years and above 18 years, students unwilling to participate, and students whose parents did not give their consent for participation were excluded from the survey. Eligible students were stratified and randomised proportionally in the schools depending on the class sizes and a total of 2068 students were selected from the fifteen schools.

Self-administered questionnaires were distributed to eligible respondents on voluntary and anonymous bases for the data collection after a pilot study. The questionnaire was designed in English because it is the language of instruction in schools in the study area. However, the interpretation of items in the main dialect in the region was also given to respondents by the research assistant on individual needs. The questionnaire administration took an average of 45 min in each school. Participants were compensated with stationary for their time. All ethical requirements were observed as indicated in the ethics approval section of this paper.

### Measures

#### Familial social capital

The domains of familial social capital used in this study were adopted from Morgan et al.’s [3] social capital framework. Two sub-domains of social capital - “sense of belonging (identity and safety with their environment), autonomy and control (perceptions of power to influence decisions)” [32] included in this framework have been rigorously assessed as indicators of young people’s social capital and as vital ‘health assets’ for promoting wellbeing for young people [2–4]. These sub-domains ‘sense of belonging’ and ‘autonomy and control’ have also been found to be significant ‘health assets’ for the life satisfaction and happiness of adolescents in the sub-Saharan African context [21]. Considering the social context, familial social capital was examined in terms of family sense of belonging (FSB) and family autonomy and control (FAC) which reflect young people’s psychological health and wellbeing, as well as cognitive social capital. Family autonomy and control were measured separately as family autonomy support (FAS) and family control (FC) since they are regarded as separate constructs [21]. FAS and FC were measured using scales adopted from Marbelle & Grolnick’s [33] 18-item parental autonomy and 9-item control scales respectively. These scales were developed by Marbelle & Grolnick [33] using Ghanaian young people and were reliable in this study. The reliability of the FAS and FC scales from this study was α = .87 and α = .73 respectively. The items for FAS and FC were scored as: not true at all (1); not true (2); true (3); very true (4), don’t know (5) [33]. FSB was measured by four questions; ‘how much do you feel your family understands you?’; ‘how much do you feel you and your family have fun together?’; ‘to what extent do you feel your family pays attention to you?’ and ‘how much do you feel safe at home?’ [21]. Responses were scored as follows: 1 = very little, 2 = somewhat, 3 = neutral, 4 = quite a bit, 5 = very much and 6 = don’t know. The FSB scale in this study was reliable to measure Ghanaian adolescents’ family sense of belonging (α = .74). Composite scores were created for all the measures of social capital (FSB, FAS, and FC). The ‘don’t know’ option and those with missing data for any of the measures were not scored when creating the composite scores. Based on the quartile values obtained from each of the three composite scores of social capital (FSB, FAS, and FC), three categories (low, medium, and high) were created using SPSS descriptive statistics to access variations in the respondents’ amount of social capital. For instance, for family autonomy support, there were five responses which were scored as follows: not true at all (1); not true (2); true (3); very true (4) and (5) ‘don’t know’ was not scored. The scores ranged from 18 to 72 and were grouped using quartile values in SPSS as low FAS (18–42); medium (43–52); high (53–72) FAS. Other quartile ranges were: Low FSB = 4–12, medium FSB = 13–18, high FSB =19–20, low FC = 9–21, medium FC = 22–36 and high FC = 27–36. A high score for FSB and FAS represented high social capital while a high score for FC represented low social capital because ‘control’ is regarded as a negative facet of social capital [15].

#### Socioeconomic factor

One critical socioeconomic variable of young people, material affluence (MA) was used and measured using Doku et al.’s [34] material affluence scale which has been shown to be a reliable measure for Ghanaian adolescents socioeconomic status [21, 34]. MA was measured by developing a composite score from eight items of two broad types; household assets (television, fridge, computer, radio, electricity, family car, and own room) and housing characteristics (blockhouse and non-block house). Responses to having these items in their homes were scored as Yes = 1 and No = 0, the reliability of the scale in this study was α = .703. The ‘don’t know’ option and those with missing data were not scored. Three levels (low, medium, and high) of MA were created based on the quartile values obtained from the composite score to assess variations in the respondents’ socioeconomic background. Using these eight items, the scores ranged from 0 to 8 and were grouped as low SES (0–3); medium SES (4–5), and high SES (6–8).

#### Demographic factors (DFs)

This study employed adolescents of 13-18 years old. Seven demographic characteristics were employed as measures of demographic factors which included gender (1 = male, 2 = female), age (collapsed into two cohorts (1 = young adolescents (13-14 yrs), 2 = older adolescents (15-18 yrs)), and religion (1 = Christian, 2 = traditionalist, 3 = Muslim; traditionalist was excluded in the logistic regression due to the negligible sample size (0.9%) which yielded no result in the analysis output). Others include ethnicity (1 = Waala, 2 = Dagaaba, 3 = Sissala, 4 = Northern tribe, 5 = other, 6 = Brifour), marital status (1 = married, 2 = other, 3 = separated, 4 = cohabiting, 5 = never married), educational level was derived from collapsing class levels into two variables ((1 = basic (JHS1 and JHS 2), 2 = secondary school (SHS 1and SHS 2)) and family structure (respondents were asked who they were living with; 1 = both parents, 2 = other, 3 = stepparents, 4 = family relatives, 5 = single parents, ‘other’ was excluded in the logistic regression due to its negligible sample size (0.5%)) yielding no result in the analysis output.

### Data analysis

Composite scores were created for the social capital and socioeconomic variables. The ‘don’t know’ option and those with missing data for all the measures were not scored when creating the composite scores. Three levels of social capital were created using the quartile values of the composite scores to represent different amounts of social capital. This is to examine how different segments of adolescents fall within the top, middle, and bottom of the ‘familial social capital ladder’. Univariate analysis using descriptive statistics was employed to describe the sample characteristics. Mean and standard deviation and count and percentages were used to represent continuous variables and categorical variables respectively. Bivariate analysis using the Chi-square test (χ^2^) was conducted to identify variations in the levels of social capital (FSB, FAS, and FC) possessed by the respondents with respect to their material affluence and demographic factors. Finally, a series of multivariate analysis using multinomial logistic regression models were employed to determine the relationships between material affluence and demographic factors, and the levels of social capital. Multinomial regression was used to also observe the odds of different segments of adolescents to achieve medium and high amounts of social capital than to achieve low amounts of social capital. Since the aim of the study was not to examine independent contributions of either material affluence or sociodemographic factors, these predictors were employed in the same models as adjusted models. Three separate adjusted models (Model 1, Model 2, and Model 3) containing material affluence and all the demographic factors were hence employed against each of the three indicators of social capital (family sense of belonging – Model 1; family autonomy support- Model 2 and family control- Model 3). Analyses were conducted using IBM-SPSS for Windows application (version 23.0) software and the level of significance was *p* < 0.05 (two-tailed). Estimated OR and 95% Wald’s confidence interval (CI) were employed as measures of predictor effects.

## Results

Table 1 shows the characteristics of the respondents of which 2068 adolescents participated in the study survey. There were no missing data for both reported material affluence and demographic factors. However, the adolescents could not report fully their family sense of belonging, family autonomy support, and family control measures, hence, there were some missing data for these measures (see Table 1). Participants with missing data were excluded when creating composite scores. The study analysis, hence, involved a number of 1909, 1497, and 1730 adolescents in the analysis relating to family sense of belonging (FSB), family autonomy support and family control (FC) respectively (see Tables 1 and 2). Table 2 indicates that about 34, 32, and 21.9% of males reported high FSB, high FAS, and high FC respectively. By contrast, about 28, 23, and 31% of females reported high FSB, high FAS, and high FC respectively. Also, about 26, 23 and 31% of adolescents from low affluence background (MA) evaluated their FSB, FAS, and FC as high respectively, while, about 41, 38 and 21% of adolescents from high affluence background evaluated their FSB, FAS, and FC as high respectively (See Table 2). Thus, Table 2 shows significant variations in FSB, FAS and FC by socioeconomic and demographic factors. From Table 3, adolescents from low affluence households had about 63 and 61% lower odds of attaining a high family sense of belonging (FSB) (OR = 0.373; 95%CI: 0.271–0.513) and high family autonomy support (FAS) (OR = 0.387; 95%CI: 0.270–0.556) than attaining low FSB and low FAS respectively but had 67% higher odds of reporting high family control (FC) than reporting low FC (OR = 1.673; 95%CI: 1.187–2.359) than their counterparts. Males had about 55 and 71% higher odds to possess high FSB (OR = 1.549; 95%CI: 1.210–1.983) and high FAS (OR = 1.705; 95%CI: 1.272–2.284) respectively but had 38% lower odds to reporting high family control (OR = 0.624; 95%CI: 0.474–0.822) than females. The odd of young adolescents to possess high FSB than older adolescents was about 66% higher (OR = 1.662; 95%CI: 1.168–2.367). The odd of secondary school adolescents to possess high FSB than basic school adolescents was about 232% higher (OR = 3.323; 95% CI: 2.322–4.757). There were other demographic factors that predicted familial social capital (see Table 3).

**Table 1:** Participants characteristics

	Valid N	(%)	Mean (SD) (Range)
**Variables**			
**Personal Characteristics**			
**Age**			16.25 (±1.492) (13-18 years)
**Age cohort**			
Young adolescent	600	(29.01)	
Older adolescent	1468	(70.99)	
**Gender**			
Male	988	(47.8)	
Female	1080	(52.2)	
**Educational level**			
Secondary	1394	(67.4)	
Basic	674	(32.6)	
**Family structure**			
Both parents	1262	(61.0)	
Other	10	(0.5)	
Stepparents	108	(5.2)	
Family relatives	271	(13.1)	
Single parent	417	(20.2)	
**Ethnicity**			
Waala	266	(12.9)	
Dagaaba	1252	(60.5)	
Sissala	96	(4.6)	
Northern tribe	188	(9.0)	
Other	118	(5.7)	
Brifour	148	(7.2)	
**Marital status**			
Married	65	(3.1)	
Other	15	(0.7)	
Separated / broke-up	18	(0.9)	
Cohabiting	65	(3.1)	
Never married	1905	(92.1)	
**Religion**			
Christian	1501	(72.6)	
Muslim	548	(26.5)	
Traditionalist	19	(0.9)	
**Material affluence**			
Low	853	(41.2)	
Medium	667	(32.3)	
High	548	(26.5)	
**Family sense of belonging**			
Low	563	27.2	
Medium	764	36.9	
High	582	28.1	
Missing	159	7.7	
**Family autonomy support**			
Low	385	18.6	
Medium	687	33.2	
High	407	19.7	
Missing	589	28.5	
**Family control**			
Low	438	21.2	
Medium	833	40.3	
High	459	22.2	
Missing	338	16.3	

*N* Sample size, *%* sample percentage, *SD* Standard deviation

**Table 2:** Bivariate results of socioeconomic, demographic factors and familial social capital of adolescents in Ghana using cross-tabulation- Chi-square

Variables	Level of Family Sense of belonging ***N*** = 1909						χ^**2**^	Level of Family autonomy support ***N*** = 1479						χ^**2**^	Level of Family control ***N*** = 1730						χ^**2**^
	Low		Medium		High			Low		Medium		High			Low		Medium		High		
**Gender,**	N	(%)	N	(%)	N	(%)		N	(%)	N	(%)	N	(%)		N	(%)	N	(%)	N	(%)	
Male	235	(25.5)	377	(40.9)	310	(33.6)	15.779***	170	(23.3)	329	(45.1)	230	(31.6)	13.090**	223	(27.1)	420	(51.0)	180	(21.9)	17.521***
Female	328	(33.2)	387	(39.2)	272	(27.6)		215	(28.7)	358	(47.7)	177	(23.6)		215	(23.7)	413	(45.5)	279	(30.8)	
Total	**563**	**(29.5)**	**764**	**(40.0)**	**582**	**(30.5)**		**385**	**(26.0)**	**687**	**(46.5)**	**407**	**(27.5)**		**438**	**(25.3)**	**833**	**(48.2)**	**459**	**(26.5)**	
**Age cohort**																					
Young adolescent	167	(31.0)	235	(43.6)	137	(25.4)	9.301*	96	(25.7)	177	(47.5)	100	(26.8)	0.216	95	(20.2)	214	(45.4)	162	(34.4)	22.848***
Older adolescent	396	(28.9)	529	(38.6)	445	(32.5)		289	(26.1)	510	(46.1)	307	(27.8)		343	(27.2)	619	(49.2)	297	(23.6)	
Total	**563**	**(29.5)**	**764**	**(40.0)**	**582**	**(30.5)**		**385**	**(26.0)**	**687**	**(46.5)**	**407**	**(27.5)**		**438**	**(25.3)**	**833**	**(48.2)**	**459**	**(26.5)**	
**Educational level**																					
Secondary	329	(25.2)	510	(39.1)	464	(35.6)	61.180***	286	(26.1)	500	(45.7)	309	(28.2)	1.329	334	(27.3)	597	(48.9)	291	(23.8)	18.688***
Basic	234	(38.6)	254	(41.9)	118	(19.5)		99	(25.8)	187	(48.7)	98	(25.5)		104	(20.5)	236	(46.5)	168	(33.1)	
Total	**563**	**(29.5)**	**764**	**(40.0)**	**582**	**(30.5)**		**385**	**(26.0)**	**687**	**(46.5)**	**407**	**(27.5)**		**438**	**(25.3)**	**833**	**(48.2)**	**459**	**(26.5)**	
**Family structure**																					
Both parents	292	(25.2)	483	(41.7)	382	(33.0)	31.825***	218	(24.6)	416	(47.0)	252	(28.4)	7.500	278	(26.2)	510	(48.0)	275	(25.9)	19.680*
Other	3	(30.0)	5	(50.0)	2	(20.0)		3	(37.5)	5	(62.5)	0	(0.0)		2	(20.0)	5	(50.0)	3	(30.0)	
Stepparent	45	(43.3)	33	(31.7)	26	(25.0)		20	(25.3)	40	(50.6)	19	(24.1)		12	(13.2)	39	(42.9)	40	(44.0)	
Family relatives	84	(33.6)	103	(41.2)	63	(25.2)		57	(31.0)	77	(41.8)	50	(27.2)		55	(25.2)	115	(52.8)	48	(22.0)	
Single parent	139	(35.8)	140	(36.1)	109	(28.1)		87	(27.0)	149	(46.3)	86	(26.7)		91	(26.1)	164	(47.1)	93	(26.7)	
Total	**563**	**(29.5)**	**764**	**(40.0)**	**582**	**(30.5)**		**385**	**(26.0)**	**687**	**(46.5)**	**407**	**(27.5)**		**438**	**(25.3)**	**833**	**(48.2)**	**459**	**(26.5)**	
**Religion**																					
Christian	405	(29.1)	562	(40.4)	425	(30.5)	8.811	267	(24.3)	523	(47.6)	309	(28.1)	9.580*	308	(24.6)	609	(48.7)	333	(26.6)	8.163
Traditionalist	11	(61.1)	4	(22.2)	3	(16.7)		7	(50.0)	5	(35.7)	2	(14.3)		0	(0.0)	9	(56.3)	7	(43.8)	
Muslim	147	(29.5)	198	(39.7)	154	(30.9)		111	(30.3)	159	(43.4)	96	(26.2)		130	(28.0)	215	(46.3)	119	(25.6)	
Total	**563**	**(29.5)**	**764**	**(40.0)**	**582**	**(30.5)**		**385**	**(26.0)**	**687**	**(46.5)**	**407**	**(27.5)**		**438**	**(25.3)**	**833**	**(48.2)**	**459**	**(26.5)**	
**Marital status**																					
Married	22	(37.9)	24	(41.4)	12	(20.7)	12.469	7	(14.3)	26	(53.1)	16	(32.7)	11.985	14	(25.9)	24	(44.4)	16	(29.6)	1.914
Other	3	(23.1)	3	(23.1)	7	(53.8)		6	(50.0)	3	(25.0)	3	(25.0)		2	(25.0)	4	(50.0)	2	(25.0)	
Separated/ broke-up	6	(37.5)	7	(43.8)	3	(18.8)		6	(46.2)	5	(38.5)	2	(15.4)		2	(14.3)	7	(50.0)	5	(35.7)	
Cohabiting	13	(20.0)	25	(38.5)	27	(41.5)		13	(24.1)	22	(40.7)	19	(35.2)		14	(26.4)	27	(50.9)	12	(22.6)	
Never married	519	(29.5)	705	(40.1)	533	(30.3)		353	(26.1)	631	(46.7)	367	(27.2)		406	(25.4)	771	(48.2)	424	(26.5)	
Total	**563**	**(29.5)**	**764**	**(40.0)**	**582**	**(30.5)**		**385**	**(26.0)**	**687**	**(46.5)**	**407**	**(27.5)**		**438**	**(25.3)**	**833**	**(48.2)**	**459**	**(26.5)**	
**Ethnicity**																					
Waala	57	(23.8)	102	(42.5)	81	(33.8)	16.896	36	(22.4)	88	(54.7)	37	(23.0)	16.738	65	(30.8)	109	(51.7)	37	(17.5)	28.466**
Dagaaba	338	(29.2)	464	(40.1)	354	(30.6)		231	(25.4)	416	(45.7)	264	(29.0)		251	(23.9)	505	(48.0)	296	(28.1)	
Sissala	33	(37.1)	29	(32.6)	27	(30.3)		25	(33.8)	34	(45.9)	15	(20.3)		19	(23.2)	33	(40.2)	30	(36.6)	
Northern tribe	53	(31.0)	66	(38.6)	52	(30.4)		35	(27.3)	60	(46.9)	33	(25.8)		44	(28.6)	71	(46.1)	39	(25.3)	
Other	28	(25.2)	44	(39.6)	39	(35.1)		21	(21.4)	42	(42.9)	35	(35.7)		39	(35.5)	50	(45.5)	21	(19.1)	
Brifour	54	(38.0)	59	(41.5)	29	(20.4)		37	(34.6)	47	(43.9)	23	(21.5)		20	(16.5)	65	(53.7)	36	(29.8)	
Total	**563**	**(29.5)**	**764**	**(40.0)**	**582**	**(30.5)**		**385**	**(26.0)**	**687**	**(46.5)**	**407**	**(27.5)**		**438**	**(25.3)**	**833**	**(48.2)**	**459**	**(26.5)**	
**Material affluence**																					
Low	267	(34.4)	312	(40.2)	198	(25.5)	56.223***	183	(29.6)	295	(47.7)	140	(22.7)	36.103***	154	(21.6)	342	(47.9)	218	(30.5)	17.150**
Medium	202	(32.9)	238	(38.8)	174	(28.3)		121	(26.8)	220	(48.8)	110	(24.4)		145	(26.6)	259	(47.5)	141	(25.9)	
High	94	(18.1)	214	(41.3)	210	(40.5)		81	(19.8)	172	(42.0)	157	(38.3)		139	(29.5)	232	(49.3)	100	(21.2)	
Total	**563**	**(29.5)**	**764**	**(40.0)**	**582**	**(30.5)**		**385**	**(26.0)**	**687**	**(46.5)**	**407**	**(27.5)**		**438**	**(25.3)**	**833**	**(48.2)**	**459**	**(26.5)**	

***p < 0.001, **p < 0.005, *p < 0.05, *N = sample size*

**Table 3:** Multinomial regression (Adjusted ORs and 95% CI) for the relationship between socioeconomic, demographic factors and familial social capital of adolescents in Ghana

	Models 1^**a**^				Model 2^**b**^				Model 3^**c**^			
	Medium FSB		High FSB		Medium FAS		High FAS		Medium FC		High FC	
	OR	95%C.I	OR	95%C.I	OR	95%C.I	OR	95%C.I	OR	95%C.I	OR	95%C.I
**Independent variables**												
Gender (ref: female)												
Male	1.349	(1.074–1.694)*	1.549	(1.210–1.983)**	1.166	(0.899–1.513)	1.705	(1.272–2.284)***	0.962	(0.760–1.218)	0.624	(0.474–0.822)**
Age cohort (ref: older adolescents)												
Young adolescents	1.571	(1.151–2.144)**	1.662	(1.168–2.367)**	1.078	(0.735–1.581)	1.106	(0.717–1.708)	1.160	(0.821–1.639)	1.596	(1.089–2.338)*
Educational level (ref: basic)												
Secondary	1.712	(1.259–2.329)**	3.323	(2.322–4.757)***	0.823	(0.555–1.220)	0.886	(0.566–1.388)	1.010	(0.714–1.430)	0.820	(0.556–1.211)
Family structure (ref: single parent)												
Both parents	1.514	(1.139–2.012)**	1.549	(1.137–2.110)**	1.023	(0.741–1.413)	0.972	(0.675–1.399)	1.059	(0.783–1.431)	1.077	(0.763–1.521)
Stepparents	0.712	(0.419–1.211)	0.919	(0.514–1.641)	0.897	(0.479–.1.680)*	0.762	(0.369–1.574)	1.706	(0.840–3.464)	2.783	(1.339–5.784)*
Family relatives	1.164	(0.792–1.709)	0.996	(0.644–1.540)	0.687	(0.439–1.074)	0.708	(0.427–1.174)	1.188	(0.781–1.806)	0.857	(0.519–1.415)
Religion (ref: Muslim)												
Christianity	1.134	(0.821–1.564)	1.209	(0.849–1.723)	1.961	(1.348–2.854)***	1.447	(0.961–2.178)	1.076	(0.768–1.507)	0.849	(0.581–1.240)
Marital status (ref: never married)												
Married	0.739	(0.401–1.361)	0.395	(0.184–0.848)*	2.300	(0.927–5.708)	2.260	(0.861–5.932)	0.875	(0.443–1.728)	1.173	(0.551–2.499)
Other	0.398	(0.65–2.435)	1.896	(0.468–7.681)	0.256	(0.62–1.062)	0.385	(0.090–1.651)	0.788	(0.129–4.807)	0.863	(0.116–6.419)
Separated / broke-up	0.968	(0.298–3.145)	0.559	(0.125–2.498)	0.395	(0.102–1.521)	0.324	(0.060–1.735)	1.811	(0.372–8.817)	2.031	(0.360–11.461)
Cohabiting	1.149	(0.576–2.295)	1.422	(0.708–2.853)	0.916	(0.451–1.861)	1.243	(0.593–2.606)	1.094	(0.563–2.127)	1.087	(0.484–2.439)
Ethnicity (ref: Brifour)												
Waala	1.431	(0.807–2.539)	1.920	(0.986–3.738)*	3.476	(1.755–6.885)***	1.816	(0.817–4.040)	0.632	(0.327–1.222)	0.382	(0.177–0.823)*
Dagaaba	1.079	(0.711–1.639)	1.461	(0.874–2.441)	1.492	(0.916–2.430)	1.751	(0.983–3.118)	0.691	(0.404–1.181)	0.826	(0.455–1.500)
Sissala	0.669	(0.336–1.331)	0.901	(0.417–1.944)	1.808	(0847–3.857)	1.200	(0.482–2.990)	0.646	(0.288–1.446)	1.206	(0.507–2.865)
Northern tribes	0.937	(0.541–1.622)	1.235	(0.651–2.344)	1.704	(0.896–3.241)	1.641	(0.775–3.471)	0.552	(0.290–1.053)	0.606	(0.291–1.260)
Other	1.051	(0.548–2.016)	1.300	(0.627–2.698)	1.883	(0.908–3.908)	2.500	(1.118–5.593)	0.502	(0.253–0.997)*	0.451	(0.199–1.021)
Socioeconomic status (ref: high)												
Low	0.549	(0.405–0.743)***	0.373	(0.271–0.513)***	0.775	(0.554–1.085)	0.387	(0.270–0.556)***	1.262	(0.942–1.692)	1.673	(1.187–2.359)**
Medium	0.546	(0.398–0.748)***	0.442	(0.318–0.615)***	0.866	(0.607–1.237)	0.472	(0.322–0.693)***	1.024	(0.759–1.382)	1.190	(0.831–1.704)
N	1881				1457				1704			
*Model fitting information*												
2Log-likelihood	− 1714.550				− 1400.445				− 1588.281			
Nagelkerke Pseudo-*R*^2^	0.099				0.072				0.061			

***p < .001; **p ≤ .005; *p < .05

^a^ = adjusted model comprising material affluence (MA), demographic factors (DFs) (gender, age, educational level, family structure, religion, marital status, ethnicity), and family sense of belonging (FSB). ^b^ = adjusted model comprising MA, DFs (gender, age, educational level, family structure, religion, marital status, ethnicity) and family autonomy support (FAS). ^c^ = adjusted model comprising MA, DFs (gender, age, educational level, family structure, religion, marital status, ethnicity), and family control (FC). The last categories were used as the reference categories for MA and all the DFs. The low category was used as a reference for FSB, FAS, and FC. ‘ref’ = reference

## Discussion

This study found adequate evidence to propose that socioeconomic and demographic factors are independent determinants of adolescent’s familial social capital. This has been accomplished through the examination of the relationship between material affluence, several demographic factors, and several indicators of adolescents’ cognitive social capital concomitantly. This approach which has not been employed by many researchers offers uniqueness to this study as it provides insight into how material affluence and various demographic factors are related to different sub-domains of young people’s cognitive social capital in the sub-Saharan African context.

### Family sense of belonging (FSB)

This study found significant variations in the adolescents’ family sense of belonging by gender, age cohort, educational level, family structure, and material affluence. Consistent with previous studies [20, 35, 36], this study found that adolescents from high socioeconomic backgrounds were protected against a low family sense of belonging compared to those from a low socioeconomic background. Poor adolescents hence have lower levels of this domain of familial social capital. Also, ‘a male adolescent’ offered a greater opportunity for a high family sense of belonging compared to ‘a female adolescent’. This is contrary to the findings of the United States’ adolescents [25]. The finding reflects the male dominancy culture of the study region and the African socio-cultural hegemony which prioritises male children by families over female children [37, 38]. This contradiction in findings, hence, informs the demographic and contextual nature of social capital. Moreover, similar to other studies, this study found that the family sense of belonging of the study adolescents declined with age. This is possibly because older adolescents tend to have a lesser close relationship with parents and parental involvement declines with age [39, 40]. Furthermore, adolescents in secondary schools tended to have higher FSB than those in basic schools. Considering that all the respondents in secondary schools were residing in school dormitories away from their families, it is likely that their residential status might have protected them from constant ‘adolescence - family conflicts’ [41] compared to the adolescents from basic schools who were residing with their families during the period of this study survey.

Additionally, adolescents from both biological parent households were protected against a low family sense of belonging compared to adolescents from single-parent households. There is, therefore, the possibility that belonging to both biological parent households provides additional opportunities for adolescents to accumulate more socioemotional resources; providing prospects for a high sense of belonging [42]. Again, contrary to Nieminen et al., [28], our study found that married adolescents were more vulnerable to low levels of social capital, low family sense of belonging. This contradiction in findings on marital status can possibly be related to the negative psychological effect of child marriage [43]. Contrary to King & Boyd [26], religion played no significant role in the family sense of belonging of adolescents in this study. This is perhaps because, generally, the respondents were all religious with most being Christians and Muslims. Finally, ethnicity triggered inequality in the family sense of belonging of the respondents. This finding can possibly be linked to the socioeconomic conditions of the various ethnic groups in the study area as ethnic groups are usually allocated in particular poverty zones.

### Family autonomy support (FAS) and family control (FC)

The study has revealed variations in family autonomy support by gender, religion, and material affluence while revealing variations in family control by gender, age cohort, educational level, family structure, ethnicity, and material affluence. The present analysis shows that belonging to families with high socioeconomic backgrounds safeguarded adolescents against low family autonomy support and high family control. These findings echo the effects of socioeconomic status on parenting styles [44, 45]. Again, gender was a significant ‘culprit’ for inequalities in the level of family autonomy support and family control of this study’s adolescents. This finding is supported by existing literature that suggests that parents are biased in their parenting styles based on the child’s gender [46–48]. Similar to claims by Domenech et al. [46] and Ahl et al., [47], male adolescents were more likely to “perceive power to influence decision” than females. These findings underscore the male dominancy culture of the study setting which probably provides more opportunities for males to participate in decision-making processes compared to the females. Moreover, from this study, religion was significant for establishing inequality in family autonomy support but not for family control. Muslim adolescents appeared to be granted low participation in familial decision-making compared to the remaining religious groups. However, no literature was found to support this claim and therefore it is difficult to explain. However, it is probable that this finding is peculiar to the Ghanaian context. Furthermore, according to the findings, while age was not a significant factor for family autonomy support, the respondents’ family control declined with age. This is likely due to children’s increase in self-control over time [49] which possibly decreased family control of the older adolescents. Also, the study revealed that adolescents from stepparent households experienced lower family autonomy support and higher family control than their counterparts. This finding can be linked to the poor relationship between stepchildren and their stepparents which results in tenacity for the child to resist the authority of the stepparent [39, 50]. Ethnicity was also responsible for inequalities in the level of family autonomy support and family control of the study’s adolescents. These findings can also be linked to the socioeconomic setting of the ethnic groups which accentuate the effect of socioeconomic status on parental behaviours towards their children. These findings indicate that poor adolescents have lower levels of this domain (FAC) of familial social capital. Lastly, all the determinants related differently with family autonomy support and family control which supports the claim that parental autonomy support and parental control are distinct constructs of parenting styles [21].

### Strengths and limitations

The study employed sub-domains of social capital that were developed as part of the World Health Organisation-Health Behaviour in School-aged Children (WHO-HBSC) optional package. These sub-domains have been widely validated and confirmed in cross-national studies to be protective health assets for young people [3, 4]. Employing these sub-domains of social capital to in-school adolescents in this study may potentially provide a strong base for public health policy in especially sub-Saharan Africa to acknowledge the significance of the findings from this study even from a global perspective. Secondly, this study adopted reliable measurement scales that were developed purposely for measuring the socioeconomic status and parental autonomy and control of young people in Ghana as well as employed a reliable scale for measuring the family sense of belonging of young people in Ghana. This study, therefore, presents a true depiction of the socioeconomic background and cognitive social capital of young people from Ghana. Thirdly, this study is one of the few to investigate the role of socioeconomic and demographic factors in establishing inequalities in young people’s cognitive and familial social capital in a sub-Saharan African context. Moreover, this study is one of the few to consider the effect of material affluence on young people’s familial social capital as many studies, especially from sub-Saharan Africa, have focused on family educational level, family income, and employment status to assess young people’s socioeconomic factors. This study may, therefore, provide a guide to future research on promoting the familial social capital of young people in sub-Saharan Africa. The limitation of this study includes the use of cross-sectional data which did not allow any causal relationships between material affluence, demographic factors, and social capital to be established. Secondly, despite the representative sample used, only adolescents from Northern Ghana were included in the study. However, the findings may proffer first-hand evidence and knowledge base for future research on this important but largely neglected topic. Future studies should employ a national analysis to allow for a strong generalisation of the findings.

## Conclusions

This study has provided significant insight into the role of socioeconomic and demographic factors in establishing inequalities in familial social capital among young people in the sub-Saharan African context-Ghana. Material affluence, gender, age, educational level, religion, family structure, marital status, and ethnicity are crucial determinants of young people’s familial social capital. Gender and material affluence appear to present the greatest inequalities in young people’s familial social capital with females and poor adolescents being left behind in all dimensions of familial ‘health assets’. Additionally, the respondents’ level of cognitive social capital reflects the level of social and psychological health and wellbeing of young people in Ghana. The family context, therefore, seems to be failing some cohorts of young people in Ghana concerning their wellbeing. Public health policies should address socioeconomic and demographic barriers to young people’s accumulation of social capital and design targeted policies that would protect the cohorts of young people at risk of familial social capital inequality. The role of the family in constructing social capital for young people’s wellbeing should be promoted and an integrated approach with families to social capital promotion strategies for young people should be adopted. Finally, there are variations in how socioeconomic and demographic factors affect various sub-domains of young people’s familial social capital in Ghana. Researchers, particularly, in sub-Saharan Africa, therefore, need to ensure a holistic assessment comprising a wide array of socioeconomic and demographic factors and several dimensions of young people’s familial social capital for a comprehensive policy contribution.

## Data Availability

The datasets used and/or analysed during the current study are available from the corresponding author on reasonable request.

## Abbreviations

DFs: Demographic factors
FAS: Family autonomy support
FAC: Family autonomy and control
FC: Family control
FSB: Family sense of belonging
MA: Material affluence
